# A Novel Paramyxovirus?

**DOI:** 10.3201/eid1101.040653

**Published:** 2005-01

**Authors:** Christopher F. Basler, Adolfo García-Sastre, Peter Palese

**Affiliations:** * Mount Sinai School of Medicine, New York, New York, USA

**Keywords:** angiotensin II, fusion protein, kidney, matrix protein, mesangial cell, paramyxovirus, phosphoprotein, polymerase chain reaction, dispatch

## Abstract

In public databases, we identified sequences reported as human genes expressed in kidney mesangial cells. The similarity of these genes to paramyxovirus matrix, fusion, and phosphoprotein genes suggests that they are derived from a novel paramyxovirus. These genes are sufficiently unique to suggest the existence of a novel paramyxovirus genus.

The identification of novel viruses, particularly those with the potential to cause human disease, has important public health and scientific implications. Examples of emerged or recently identified viruses affecting humans include HIV (*1*), hepatitis C virus (*2*), West Nile virus (*3*), severe acute respiratory syndrome (SARS)-associated coronavirus (*4*), human coronavirus from the Netherlands (HCoV-NL)(*5*), and Ebola virus (*6*). Among the paramyxoviruses, Nipah virus, Hendra virus, and human metapneumovirus were recently described as causing disease in humans (*7*–*9*).

We now report as viral sequences nucleotide sequences that were previously described to be human genes, named Angrem52 and Angrem104. These genes’ expression in human kidney mesangial cells was reportedly upregulated by treatment with angiotensin II (*10*,*11*). However, these genes appear to encode viral proteins with striking homology to those of paramyxoviruses. A careful analysis of these sequences suggests that they actually belong to the *Paramyxoviridae* and represent a novel genus in this virus family. Given the identification of these putative “orphan paramyxovirus” (putative OPmV) sequences from human cells, the putative OPmV may cause human infections.

## The Study

**Figure 1:**
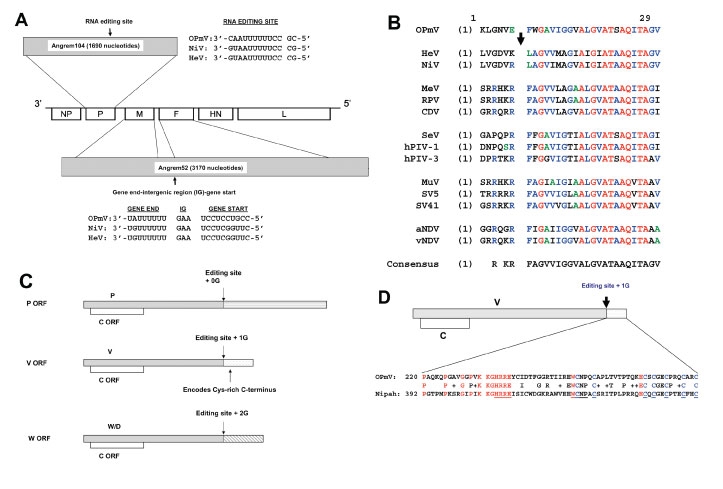
Angrem52 and Angrem104 appear to be paramyxovirus genes. A) Gene positions of a generic paramyxovirus and predicted genome position of Angrem104 (top), the phosphoprotein (P) gene, Angrem52 (bottom), the matrix protein (M) and fusion protein (F) genes. A potential editing site (nucleotides 783–795), which might allow production from the OPmV P gene of V and W/D proteins, is shown in genomic (negative) sense aligned with the proposed editing sites of Nipah virus (NC_002728) (*1*) and Hendra viruses (NC_001906). The full-length P open reading frame (ORF) was obtained by inserting an additional nucleotide in the reported Angrem104 sequence (see text). Angrem52 is predicted to be a “read-through” product of the M and F genes, a novel paramyxovirus. The full-length F ORF was obtained by making 5 changes to the reported Angrem52 sequence (see text). Putative gene-end, intergenic (IG), and gene-start transcription regulatory signals lying between OPmV M and F genes are shown aligned to the corresponding signals from Nipah and Hendra virus (shown in genomic sense [*12*]). B) The putative OPmV F protein contains a fusion peptide. The sequences surrounding the F protein cleavage sites, including most fusion peptides, of several paramyxoviruses, including putative OPmV, were aligned by using the AlignX program of the Vector NTI6 software package. The arrow indicates the cleavage site. Residues in red are absolutely conserved. Residues in blue are conserved in most sequences. C) Putative genome organization of the putative OPmV P gene, allowing translation of P, V, W, and C ORFs. D) Alignment of cysteine-rich carboxy-termini of the putative OPmV and Nipah virus V proteins. The conserved carboxy-terminal regions of the V proteins were aligned by using the AlignX program of the Vector NTI6 software package. Conserved residues are indicated in red, except for conserved cysteines, which are in blue. Underlined residues are conserved among all paramyxovirus V proteins.

A BLAST search restricted to mammalian protein sequences was performed through the NCBI Web page (http://www.ncbi.nlm.nih.gov/BLAST) by using the Nipah virus matrix protein sequence as the query. One sequence, accession number AAK76747, with homology to the Nipah virus matrix protein, was identified. This protein, called Angrem52 (for angiotensin II-induced, renal mesangial cell gene 52), displays 53% amino acid identity and 73% amino acid similarity over 337 amino acids (aa) to the Nipah virus matrix protein (data not shown). The Angrem52 protein sequence is derived from a theoretical translation of an open reading frame (ORF) from nucleotides (nt) 16 to 1038 within the 3170-nt long Angrem52 nucleotide sequence (GenBank accession no. AY040225). This notable homology suggests that Angrem52 is actually a paramyxovirus M gene (Figure 1A).

Upon further analysis of sequences within the Angrem52 cDNA downstream of the putative matrix gene, several relatively short ORFs were found to encode peptides with homology to paramyxovirus fusion (F) proteins. Modification of the reported Angrem52 sequence in several positions yields what appears to be a full-length or near full-length paramymyxovirus fusion (F) protein gene, which would be separated from the M ORF by 355 nt (Figure 1A). Specifically, the F ORF within the original Angrem52 sequence begins at position 1393 but appears to terminate prematurely with a stop codon at 2118. To obtain what appears to be a “full-length” F ORF, several modifications were made to the reported sequence in order to to incorporate the additional “F-like” sequences. An A at position 2110 was deleted. A T at position 2155 was deleted. A single nucleotide, either C or T, was added between positions 2296 and 2297. A T was deleted at 2461. The theoretical translation of this modified ORF yields a protein of 546 aa, the same length as the Nipah virus F protein (data not shown). A pairwise alignment of the resulting protein with the F protein of Nipah virus shows 32% identity and 53% similarity over 509 aa (data not shown). Within this protein, a putative fusion peptide is readily identifiable based on homology to those of other paramyxoviruses (Figure 1B). Although the cleavage site adjacent to the fusion peptide is typically a basic amino acid, the reported Angrem52 cDNA sequence has an acidic glutamic acid at this position (Figure 1B). Both Nipah and Hendra viruses possess F proteins that are cleaved at the expected site (Figure 1B) but are apparently processed by novel but ubiquitous proteases. Cleavage of these sites can occur even when the residue immediately left of the cleavage site is mutated to a nonbasic residue (A. Maisner, R.E. Dutch, pers. comm.).

Other features common to paramyxovirus fusion proteins, type I transmembrane glycoproteins, are a signal sequence, a transmembrane domain, and 2-heptad repeats. The 2- heptad repeats play an essential role in membrane fusion and are able to form trimeric coiled coils (*13*). For the putative OPmV F, a potential signal sequence from residues 1 to 23 and a potential transmembrane domain is found between residues 497 and 516. Further, the putative OPmV F has heptad repeats in the positions expected for a paramyxovirus F protein (residues 108–190 and 428–481).

Paramyxovirus genes are separated by cis-acting elements in the genome. The signals that lie between genes include a gene-end signal, an intergenic sequence, and a gene-start signal. The Angrem52 sequence, which contains the continuous sequence for the M and F genes, possesses a sequence with similarity to the regulatory sequences in other paramyxoviruses (Figure 1A).

Another reported angiotensin II-induced gene, Angrem104 (accession no. AF367870), appears to be a paramyxovirus phosphoprotein (P) gene. The reported Angrem104 sequence is 1690-nt long (*10*,*11*). The ATG that begins the P ORF is at position 90. Based on the reported sequence, an ORF is present from nt 90 to 1133, and the theoretical translation of this ORF yields a protein with homology to paramyxovirus P proteins but shorter than reported P proteins. However, the insertion of a single T residue between nucleotides at positions 1130 and 1131 results in a single reading frame that ends at position 1579 of the reported Angrem104 sequence and encodes a protein of 496 aa. Alignment of the modified protein sequence to the Nipah virus P protein shows 20.1% amino acid identity over the length of the putative OPmV protein.

Paramyxovirus P genes frequently encode multiple proteins (*13*). For example, C proteins are encoded by alternate ORFs near the 5′ end of P genes in a number of paramyxoviruses. In addition, through the process of “RNA editing,” the site-specific insertion of nontemplate encoded nucleotides by the viral polymerase, additional proteins, such as V and W proteins, can be produced (*13*). These latter proteins share amino-terminal sequences with the P proteins but differ after the editing site and thus have distinct carboxy-terminal ends (*13*). In the case of V proteins, the unique carboxy-terminus is characterized by a relatively conserved cysteine-rich domain. Analysis of the modified Angrem104 sequence identifies a C ORF (from positions 109 to 598) potentially encoding a 163-aa protein. In addition, a possible RNA editing signal similar to that found in the P gene of other paramyxoviruses is present (Figure 1A). One or 2 additional G residues added to the newly synthesized mRNA transcribed from this template sequence (i.e., the singly edited mRNA sequence would then be AAAAAAGGG) would give rise to mRNAs encoding a V or W protein (Figure 1C). The V ORF would begin at nt 90 of our modified Angrem104 sequence and end at position 964 of the modified Angrem104 sequence (this numbering does not count the additional G residue found in the edited mRNA). The carboxy-terminus of the predicted V protein is cysteine-rich, as expected for a paramyxovirus V protein (Figure 1D). The W ORF would also begin at nt 90 but would end at nt 1027 of the original Angrem104 sequence (again not including the 2 extra G nucleotides introduced by editing [Figure 1C]).

**Figure 2:**
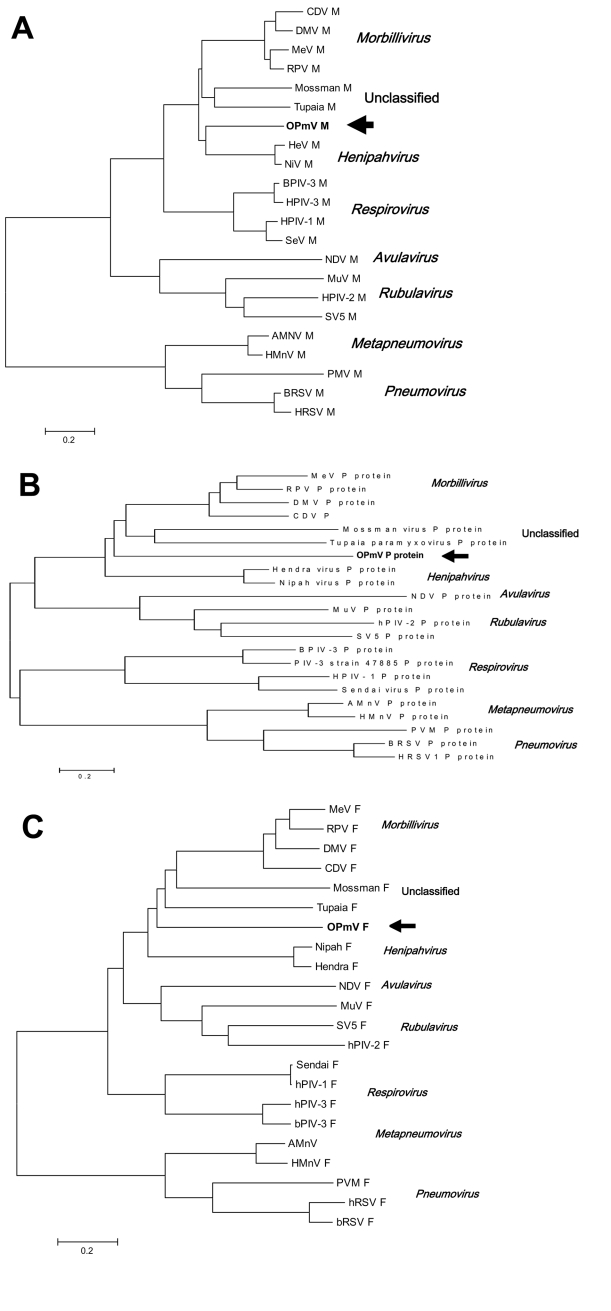
Phylogenetic comparison of OPmV proteins to other paramyxovirus proteins. A) Phylogenetic tree showing the relationship of the putative OPmV M protein to the M proteins of other paramyxoviruses representative of the various genera in the family *Paramyxoviridae*. B) Phylogenetic tree showing the relationship of the putative OPmV F protein to the F proteins of other representative paramyxoviruses. C) Phylogenetic tree showing the relationship of the putative OPmV P protein to the P proteins of other representative paramyxoviruses. Sequence alignments were made with the ClustalW method of the AlignX program of the Vector NTI6 software package. The trees were generated from these alignments by using neighbor-joining methods through the computer program MEGA version 2.1 (available from http://www.megasoftware.net/). The position of the putative OPmV sequences are indicated by arrows; distance bars, which represent 0.2 amino acid changes per position, are shown below the trees. The sequences from which the trees were constructed are as follows: Mossman, Mossman virus (NC_005339); Tupaia, Tupaia paramyxovirus (NC_002199); NiV, Nipah virus (NC_002728); HeV, Hendra virus (NC_001906); SeV, Sendai virus (AB065188); hPIV-1, human parainfluenza virus 1 (NC_003461); hPIV-3, human parainfluenza virus type 3 (NC_001796); bPIV-3, bovine parainfluenza virus type 3 (AF178655); hRSV, human respiratory syncytial virus (GI:133665); BRSV, Bovine respiratory syncytial virus (NC_001989); PMV, Pneumonia virus of mice (AY573814)AMnV, avian metapneumovirus (AY028582); HMnV, human metapneumovirus (NC_004148); SV5, simian paramyxovirus SV5 (D13868); hPIV-2, human parainfluenza virus type 2 (NC_003443); MuV, mumps virus (AY309060); NDV, Newcastle disease virus (NC_002617); MeV, measles virus (AF266288); RPV, rinderpest virus (AB021977, M21514, M34018); DMV, dolphin morbillivirus (NC_005283); CDV, canine distemper virus (NC_005283).

Based on the similarity of the predicted Angrem52 and Angrem104 sequences to paramyxovirus protein sequences, a reasonable conclusion is that Angrem52 and Angrem104 are actually paramyxovirus genes. Phylogenetic comparison of the putative OPmV P, M, and F proteins suggests that the putative OPmV belongs to a previously uncharacterized genus in the paramyxovirus family. Comparison of the M proteins may provide the most compelling argument for the uniqueness of the putative OPmV, given that an intact ORF was present in the Angrem52 nucleotide sequence and did not require additional manipulation before analysis. The putative OPmV M is found to be slightly more similar to the Henipah virus genus than to other paramyxoviruses but distinct from even Nipah and Hendra virus (Figure 2A). Likewise, the putative OPmV F protein shows the highest degree of sequence identity with the F protein of tupaia paramyxovirus (Figure 2B). Finally, analysis of the putative OPmV P gene places the putative OPmV protein on a separate branch with slightly greater similarity to the P proteins of Hendra and Nipah virus (Figure 2C). Final evidence for classifying the putative OPmV in a distinct phylogenetic group is the fact that the nucleotide sequences of its genes do not show substantial similarity to other paramyxoviruses (data not shown). Typically, notable nucleotide identity is seen between members of a paramyxovirus genus but is not seen when nucleotide sequences are compared across genera. For example, morbillivirus M genes share nucleotide identity with one another but not with the M genes of Henipah viruses (data not shown).

## Conclusions

The Angrem52 and Angrem104 genes were identified by a reverse transcriptase–polymerase chain reaction (RT-PCR)–based method from primary human mesangial cells (*10*,*11*). Basal expression of each gene was detected, but each gene was identified as an angiotensin II–induced gene (*10*,*11*). The apparent presence of viral genes in a primary human cell culture system is intriguing. The presence of these genes could reflect the presence of a virus in any of several states. These include the presence of an actively replicating, fully competent virus; the presence in the cells of a persistent virus infection; or the presence of a replicating but defective virus (*14*). Although unlikely, these genes could also reflect the integration into the cellular genome of a viral genome as cDNA (*15*,*16*).

We have performed several experiments in an effort to determine whether these paramyxoviruslike sequences are universally present within the human genome or whether they represent a very common infection found in human mesangial cells. Searches of publicly available human and mouse sequence databases have not identified proteins or predicted proteins with homology to the putative OPmV sequences (data not shown). We obtained a human 12-tissue, multiple tissue, Northern blot from Clontech and probed this with recombinant probes corresponding to the putative OPmV M and P genes. No specific signal could be obtained under conditions in which a β-actin probe efficiently recognized its mRNA (data not shown). We also screened 4 lots of Clonetics primary human mesangial cells (Cambrex, East Rutherford, NJ) for the presence of the putative OPmV mRNAs and products when the cells were untreated and after treatment with a range of concentrations of human angiotensin II (Sigma Chemical Co., St. Louis, MO). RT-PCR analysis using a number of primer sets for each of the putative OPmV genes yielded consistently negative results, and antibodies raised against recombinant forms of the putative OPmV M and P proteins failed to detect the putative OPmV proteins (data not shown). Based on these data, it appears that the putative OPmV genes are not human genes and are not universally expressed in human mesangial cells.

The source of OPmV, the genes of which were identified as Angrem52 and Angrem104, remains unclear. Infection of the cells after their establishment in culture cannot be excluded, nor can contamination of the PCR reactions used to identify Angrem52 and Angrem104 be ruled out. However, the cells may also have been infected in vivo before the generation of the primary cell culture. In this respect, the putative OPmV might be similar to simian virus 5 (SV5), which can cause persistent infection in monkey kidneys and be detected in monkeys for long periods after initial infection (*14*). Given the possibility that the putative OPmV infects human cells, the possible association of the putative OPmV with human disease is worth exploring.
